# The Association between Bodily Pain and Cognitive Impairment in Community-Dwelling Older Adults

**DOI:** 10.3390/jpm12030350

**Published:** 2022-02-25

**Authors:** Chun-Che Huang, Li-Hui Lee, Wei-Szu Lin, Tzu-Hung Hsiao, I-Chieh Chen, Ching-Heng Lin

**Affiliations:** 1 Department of Healthcare Administration, I-Shou University, Kaohsiung 82445, Taiwan; huangaj7@gmail.com; 2 Department of Medical Research, Taichung Veterans General Hospital, Taichung 40705, Taiwan; weiszu@vghtc.gov.tw (W.-S.L.); thsiao@vghtc.gov.tw (T.-H.H.); icchen@vghtc.gov.tw (I.-C.C.); 3 Department of Health Care Management, National Taipei University of Nursing and Health Sciences, Taipei 112303, Taiwan; cclhlee@ntunhs.edu.tw; 4 Department of Public Health, College of Medicine, Fu Jen Catholic University, New Taipei City 24205, Taiwan; 5 Department of Industrial Engineering and Enterprise Information, Tunghai University, Taichung 40704, Taiwan; 6 Institute of Public Health and Community Medicine Research Center, National Yang Ming Chiao Tung University, Taipei 11221, Taiwan; 7 Department of Medical Research, China Medical University Hospital, Taichung 404332, Taiwan

**Keywords:** bodily pain, cognitive impairment, pain locations

## Abstract

Background: Bodily pain is a common condition in older adults and interferes with individuals’ cognitive functioning. We aimed to evaluate the association between bodily pain and related locations and cognitive impairment among community-dwelling older adults in Taiwan. Method: In this retrospective, cross-sectional study, we enrolled 2022 participants aged 60‒70 years, from the Taiwan Biobank. Mini-Mental State Examination was performed to assess cognitive impairment. Further, logistic regression analyses were performed to identify the relationship between bodily pain and cognitive impairment. Results: Overall, 161 participants had cognitive impairment. Multivariable analysis showed that older adults who reported bodily pain were more likely than those who did not have cognitive impairment (odds ratio 1.68). Moreover, the occurrence of cognitive impairment correlated with the presence of two or more pain locations and self-reported low back and waist pain or sciatica. Conclusion: Our study revealed that cognitive impairment was associated with bodily pain in community-dwelling older adults, particularly older adults with low back and waist pain or sciatica and those with two or more pain locations. To maintain the quality of older adults’ life, pain and cognitive decline need to be simultaneously assessed with considerably more precise and objective markers.

## 1. Introduction

With the increase in life expectancy, mild cognitive impairment (MCI) and dementia are seen more commonly in older adults and continue to be an important public health concern worldwide. A pooled analysis of 11 cohort studies from the USA, Europe, Asia, and Australia showed that the prevalence of MCI was estimated between 5% and 36.7% [1]. A population-based study from Taiwan reported that approximately one-fifth of older adults (18.8%) had symptoms of MCI [2]. Contrary to dementia, MCI manifests as early memory and thinking problems in older people. It can progress to dementia, remain stable, or fully recover. Individuals with MCI and dementia who had limited daily activities may lead to an increased caregiving burden and incurred substantial healthcare and social costs [3]. Clinically, modifying risk factors, including depression and hypertension, in the early stages of MCI can prevent or delay progression to dementia [4]. The Mini-Mental State Examination (MMSE) [5] is the most often used short screening tool for providing an overall measure of cognitive impairment in community settings. Its advantage lies in the easy detection of cognitive function without direct harmful effects [6]. This brief cognitive test could be used to predict the risk of cognitive impairment among community-dwelling older adults, and to determine who requires further comprehensive assessment.

Bodily pain is not simply a widespread symptom in older adults but a complex condition with multiple causes and consequences [7]. A recent review reported that prevalence estimates of chronic pain in community-dwelling older adults ranged widely, from 25% to 76% [8]. Evidence suggested that bodily pain may potentially lead to adverse health outcomes, including disturbed sleep, impaired physical function, malnutrition, and slowed rehabilitation [7,9].

The association between chronic pain and cognitive decline in older adults has been confirmed by empirical studies [10,11,12]. Moriarty et al. highlighted a link between chronic pain and weaker performances in various cognitive functions, including memory, attention, and learning [12]. Additionally, persistent bodily pain gradually influences cognitive function [8] and leads to experiencing difficulty with daily functional abilities [8,13]. The co-occurrence of bodily pain and cognitive impairment may have an even greater impact on physical function, mental health, and daily activities than either of these symptoms alone [13,14]. Furthermore, cognitive impairment with pain is consistent with brain imaging findings, whichrevealed overlapping areas between frontal regions implicated in pain modulation and cognitive function [15], with a possible reversible effect following painful condition suppression [16].

Among the previous studies reviewed, Schepker et al. showed that pain and MCI were related to poorer mobility performance [13]. Furthermore, van der Leeuw et al. reported that whole pain severity was associated with poorer cognitive function [17]; Weiner et al. found that cross-sectionally, older adults with chronic low back pain had poorer performance than those without pain, on tests of immediate and delayed memory, learning, and mental flexibility [18]. Further, the severity of chronic low back pain was negatively correlated with neuropsychological performance [18]. However, sociocultural variables, age, and educationcould influence individual MMSE scores; thus, local standards must be developed for each population and setting evaluated [19]. The relationship between bodily pain locations and cognitive impairment has not been investigated in generally healthy older adults in Taiwan.Therefore, this study aimed to assess the association between bodily pain and related locations with cognitive impairment in community-dwelling older adults.

## 2. Materials and Methods

### 2.1. Data Source and Study Sample

The data for this retrospective, cross-sectional studywerecollected from the Taiwan Biobank, by gathering information and specimens from a convenience sample of Taiwanese volunteer participants in recruitment centers across Taiwan. The community-based database, provided by Taiwan Academia Sinica, comprised results of a questionnaire, physical examination, blood and urine tests, and experimental studies for volunteers, aged 60–70 years, with no history of cancer [20]. All data were developed and achieved by Taiwan Academia Sinica, which collected specimens and information in a complete and standardized procedure to fit researchers’ needs in different fields [20,21]. The participants’ demographic data, including age, gender, education level, marital status, health behaviors, physical activity, and family history of dementia, were recorded.

The sociodemographic data of 2022 participants aged between 60 to 70 years were retrieved from the Taiwan Biobank. The participants voluntarily completed the self-administered questionnaire and underwent physical examination and laboratory tests. All experiments were conducted in accordance with the relevant guidelines and regulations. In addition, this study was conducted in accordance with the Declaration of Helsinki, and the study protocol was approved by the Institutional Review Board of Taichung Veterans General Hospital (IRB no. CE16270B-1). All participants participated in the Taiwan Biobank cohort program voluntarily and provided written informed consent.

### 2.2. Cognitive Impairment Assessment

The primary outcome measure was the occurrence of cognitive impairment. Cognitive impairment in participants was assessed based on the Mini-Mental State Examination (MMSE), which is the most often used short screening tool for providing an overall measure of cognitive impairment in clinical, research, and community settings. MMSE cutoff scores were determined by education level, and cognitive impairment was determined according to three diagnostic thresholds: ≤16, illiterate; ≤21, less or equal to elementary school; ≤24, high school and above [22,23,24,25].

### 2.3. Bodily Pain Assessment

Bodily pain, the primary independent variable of interest, was self-reported through a complete and standardized procedure performed by the Taiwan Biobank. To ensure the accuracy of bodily pain measurement, questions were checked carefully by the well-trained interviewers and experienced auditors/reviewers who were employed by the Academia Sinica. Pain locations included articular pain, neck pain, low back and waist pain, sciatica, hemicrania continua, and others.

### 2.4. Covariates

The following covariates were evaluated: participants’ demographic (including gender, age, and marital status), personal health behaviors (including habits of alcohol drinking, smoking, and routine physical activity), physical examination (including body mass index and waist circumference), as well as blood and urine tests (including total cholesterol (TC, mg/dL), triglyceride (TG, mg/dL), high-density lipoprotein (HDL, mg/dL) cholesterol, low-density lipoprotein (LDL, mg/dL) cholesterol, fasting glucose (FG, mg/dL), and creatinine (mg/dL)).

The age of participants was divided into 60–64 years and 65–70 years. Marital status was classified into three groups: unmarried, married, and others. Status of alcohol drinking and smoking was classified into no and current use. Current use of drinking was defined as currently drinking 150 mL of alcohol per week for more than six months. Current use of smoking was defined as currently smoking for more than six months. Routine physical activity was defined by performing exercise activity at least three times per week and over 30 min each time. Body mass index (BMI) was calculated as weight (kilograms (kg)) divided by height (meters (m^2^)) squared. In addition, we used creatinine-based estimates of the glomerular filtration rate (GFR, mL/min/1.73 m^2^) as a marker for kidney function. The GFR was calculated by using the Modification of Diet in Renal Disease formula, according to the recommendation of the Taiwanese Society of Nephrology [26].

### 2.5. Statistical Analysis

All statistical analyses were performed using SAS version 9.4 software (SAS Institute Inc., Cary, NC, USA). Differences in the demographic characteristics, lifestyle, blood, and urine test results, as well as the occurrence of cognitive impairment between participants with and without bodily pain, were examined using chi-square or Fisher’s exact tests for categorical variables and Student’s *t*-test for continuous variables. Univariable and multivariable logistic regression models were performed to evaluate the bodily pain factors associated with cognitive impairment occurrence. In addition, we used bodily pain as an intermediate variable in the causal pathway analysis to identify the correlation between the variables and developing cognitive impairment. Odds ratios (ORs) with 95% confidence interval (CI) were calculated. A *p* less than 0.05 was considered to be statistically significant.

## 3. Results

### 3.1. Descriptive Statistics

The basic characteristics of study participants are shown in Table 1. A total of 2022 community-dwelling participants of older adults aged 60 to 70 years completed the questionnaire and other measures. Women reported a significantly greater percentage of bodily pain than men (56.7% vs. 43.3%, *p* < 0.001). A higher percentage of having bodily pain was observed among those who reported less or even no physical activity (*p* < 0.001), higher TG (*p* = 0.005), and GFR levels (*p* = 0.033) than among those without bodily pain, whereas they had a lower fasting glucose level (*p* = 0.006) (Table 1). However, there was no significant difference in age group, marital status, alcohol drinking, smoking, BMI, waist circumference, TC, HDL, and LDL cholesterol between the two groups.

### 3.2. Association between Bodily Pain Factors and Cognitive Impairment

Individual MMSE scores could be influenced by other factors, such as culture and education; for example, the MMSE performance among illiterates and those with low educational levels tend to bepoorer than that observed in individuals with higher education levels. In this retrospective, observational study, cognitive impairment was scored by MMSEeducation-adjusted cutoff scores. Overall, 161 (8%) of participants had cognitive impairment. Table 2 presents the results of proportions and univariable and multivariable logistic regression analyses of bodily pain factors associated with the occurrence of cognitive impairment. Self-reported bodily pain was positively correlated with cognitive impairment (75.8% vs. 65%, *p* = 0.005). The number of pain locations was positively correlated with an increased incidence of cognitive impairment (39.7% vs. 32%, *p* = 0.028). In addition, an increased proportion of cognitive impairment was observed among participants who reported low back and waist pain (39.1% vs. 30.1%, *p* = 0.021) and sciatica (16.8% vs. 10.7%, *p* = 0.026) when compared with those who did not have a cognitive impairment, respectively (Table 2).

**Figure 1:**
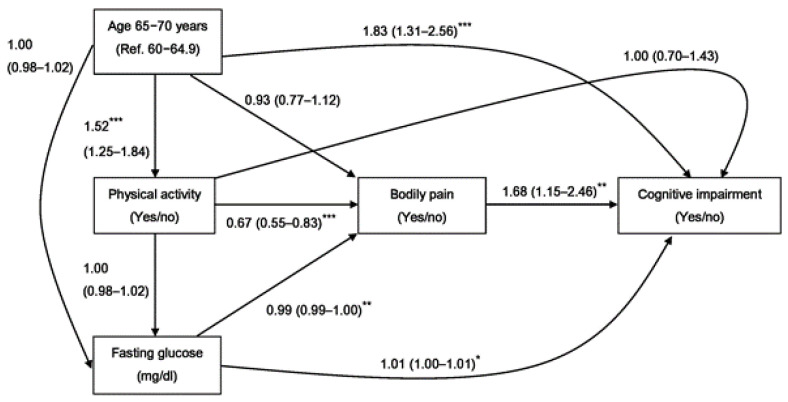
Potential causal pathway analysis between bodily pain and the occurrence of cognitive impairment among community-dwelling older adults. Values are expressed as odds ratio and 95% confidence interval. The models were adjusted for gender, marital status, alcohol drinking, smoking, physical activity, body mass index, waist circumference, total cholesterol, triglyceride, HDL cholesterol, LDL cholesterol, and eGFR by using logistic regression analyses. * *p* < 0.05, ** *p* < 0.01, *** *p* < 0.001. * = statistically significant.

Multivariable analyses showed that, after adjusting for personal demographics, health behaviors, and physical examinations, participants with bodily pain had a significantly higher probability of having a cognitive impairment (OR 1.68, 95% CI 1.15–2.46) than those without bodily pain. The odds of cognitive impairment significantly increased with the number of pain locations (OR 1.63, 95% CI 1.09–2.45). Furthermore, after the adjustments, the occurrence of cognitive impairment was greater in participants with low back and waist pain (OR 1.47, 95% CI 1.04–2.06) and sciatica (OR 1.66, 95% CI 1.06–2.59) than in those who did not, respectively (Table 2). In addition, the effect of bodily pain on cognitive impairment occurrence was associated with old age, physical activity, and fasting glucose. Figure 1 depicts the proposed causal pathway among bodily pain, predictive factors, and cognitive impairment occurrence. Physical activity and bodily pain were likely attributable to the covariates’ role as intermediate variables between advanced age and cognitive impairment development after adjusting the logistic regression model for other confounders (Figure 1).

## 4. Discussion

This study of community-dwelling older adults found that bodily pain and related locations are associated with cognitive impairment. We used a community-dwelling sample of older adults from the Taiwan Biobank to examine the relationship of bodily pain and related locations with the occurrence of cognitive impairment. As hypothesized, we found that participants of older adults aged 60 to 70 years with bodily pain experienced a significantly higher probability of having cognitive impairment than those with no bodily pain, even after adjustments for relevant covariates. This finding was consistent with prior research showing that persistent bodily pain was significantly correlated with cognitive impairment status [13]. A review of clinical and preclinical research showed that participants with chronic pain experienced a decline in a number of cognitive domains including memory, attention, and reaction time [12]. Additionally, previous studies have reportedthe detrimental effects of pain on cognition, as well as the role of age-associated changes in the brain on cognitive functions in old age; according to these studies, older adults with more pain interference had poorer memory performance [17,27]. In particular, participants with low back and waist pain or sciatica had a significantly increased risk of developing cognitive impairment when compared with those who did not. This result was consistent with a previous study for low back pain [18]. Adult patients with unrelieved low back pain and sciatica may experience a poorer prognosis with regard to cognitive, psychological function, and physical activities [8,14]. Moreover, persons with sciatica that was accompanied by numbness and motor weakness that was more severe than simple backache may develop a more intense disability [28]. Therefore, different pain locations may partially explain the observed difference in presenting cognitive impairment.

Certain older adults with chronic pain may interfere with the ability to focus their attention away from the pain locations, thereby having difficulties in responding to the cognitive tasks. Although chronic pain was associated with cognitive deficits in several cross-sectional studies in older adults, fewer studies emphasize specific pain types (e.g., neuropathic, inflammatory), irrespective of their etiology. Dick et al. compared different types of pain and found that patients’ attention was impaired to a similar level in rheumatoid arthritis, musculoskeletal pain, and fibromyalgia, compared with healthy controls [29]. Contrarily, there is evidence that cognitive functioning was worse in neuropathic pain patients than in patients with a diagnosis of mixed neuropathic and nociceptive pain [30]. Povedano et al. reported cognitive impairment in a neuropathic pain cohort, compared with the normative sample for the MMSE, and suggested that the emotional decision was more easily impaired in lumbar spinal or radicular pain of the lower back.

In the current study, the occurrence of cognitive impairment was positively associated with the number of pain locations. The older population was at risk of experiencing multisite or widespread pain [7,8,31,32]. In addition, the experience of chronic pain may shift focus from site-specific pain or pain severity to the number of locations [32]. A systematic review concluded that multiple locations and high severity of pain, and longer pain duration were recognized as potential prognostic factors for disability [33]. However, the prevalence of cognitive impairment (8%) in the present study was lower than the results of previous research [2]. This may be due to our study participants being healthy volunteers and may not be representative of all older adults with bodily pain and cognitive impairment.

Meanwhile, prior research has indicated that the number of comorbidities was positively associated with pain [34]. The pain was most frequently observed in older adults with multiple comorbidities [7,8]. Chronic pain and comorbidities both had negative effects on cognitive function [17,35]. The results of our study showed that a higher FG level was significantly associated with an increase in cognitive impairment. This finding was consistent with previous studies that indicated an association between elevated fasting blood glucose levels and cognitive impairment [36,37]. Nevertheless, other comorbid conditions were not found to be significant. Despite this, the presence of both a higher number of pain locations and comorbidities may result in much poorer cognitive status. Identifying changes in cognitive function induced by bodily pain exposure could enhance our understanding of potential biological mechanisms [37].

In multivariable logistic regression analysis, it is plausible that relationships between physical activity, bodily pain, and cognitive impairment could be bidirectional (Figure 1). In addition, previous studies have shown that physical activity induces structural and functional changes in the brain, determining enormous benefits on cognitive functioning [38]. As shown in Figure 1, the multivariable analyses in this study indicated that physical activity was associated with a decrease in the occurrence of bodily pain (OR 0.67, 95% CI 0.55–0.83). Moreover, older adults with bodily pain had a significantly higher probability of having a cognitive impairment (OR 1.68, 95% CI 1.15–2.46) than those without bodily pain. The beneficial effects of physical activity are related to increases in cerebral blood flow and maximal oxygen consumption, increasing the levels of serotonin [39,40] and beta-endorphins [40,41]. Previous studies reported that older adults with high levels of physical activity had significantly lower incidences of cognitive impairment, compared with those with a low level of physical activity [42]. However, physical activity was not significantly associated with cognitive impairment in our study. This discrepancy may be due to the lack of specific details about the intensity of physical activity. Nevertheless, the beneficial effects of physical activity should be elucidated, and physical activity should be promoted as a modifiable factor to prevent and improve cognitive decline and bodily pain.

Older adults have multiple pain-associated conditions that likely reflect multiple physiological mechanisms for bodily pain. For example, knee pain can lead to higher functional incapacity and mobility impairment than upper limb disease; therefore, future research should further assess the consequences of the differences between upper and lower limb joint pain. However, individuals do not report pain when pain sensation is absent. Therefore, continued research is required to better understand the physiological mechanisms underlying an increased cognitive impairment risk in persons with bodily pain.

Although MMSE is the weakest of cognitive measures for the detection of cognitive impairment, it is a common and easy-to-administer test for the detection of cognitive impairment in community and primary care settings; furthermore, it is more sensitive and less specific for detecting mild forms of dementia when adjusted accuracy estimates for education level [24]. Even if the MMSE has some weaknesses, it is still a useful and reliable clinical diagnostic tool, especially when used in patients with intellectual disability or deterioration for the first time. It could quickly provide data on cognitive impairment. Therefore, the current study has several noteworthy strengths. First, this study contained comprehensive information on the participants’ demographic, health behaviors, physical examination, and blood and urine test results, which had the advantage of allowing the monitoring of the health conditions and disease symptoms of community-dwelling older adults. Second, as predicted, bodily pain and related locations were significant factors affecting cognitive impairment, although pain severity was not measured in the present research. However, we used the number of pain locations as a proxy indicator for the degree of bodily pain. Third, this community-based study extended the research on identifyingbodily pain and related risk factors for impaired cognitive function.

Previous spine or other surgery, as well as the duration and intensity of pain, can have a significant influence on cognitive function, as does the nature of pain. The pain detection questionnaire can be used in community-dwelling older adults to evaluate the likelihood of pain. Further, a visual analog scale is used for the duration and intensity of pain [12]. However, data are missing on the use of painkillers, such as gabapentin, which can interfere with attention and cognitive function [12].

Our study also had some limitations. First, we examined retrospective, cross-sectional relationships and did not examine changes in bodily pain or cognitive performance over time. We did not know whether these associations have changed or not. Second, the low rate of bodily pain in survey respondents may raise issues of selection bias and generalizability to the overall older adults. The number of participants was not large enough to make any solid conclusions. However, our findings contribute to the ongoing discourse on a variety of pain conditions for community-dwelling older adults. Third, we could not exclude the potential recall bias in the assessment of bodily pain, but no further investigations were performed to confirm these data. In this study, our focus was mainly on the exclusion criteria, and the features that were tested without taking into consideration the presence of other major diseases (such as depression) that might affect the results of MMSE scores. In addition, owing to data collection limitations, our study could not differentiate between acute and chronic pain. Chronic pain is linked with depression owing to the imbalance of neuromediators in the brain [43]. Using a questionnaire for characteristics of depression could further refine our results.

## 5. Conclusions

This study revealed that an increased likelihood of occurring cognitive impairment was associated with bodily pain in community-dwelling older adults aged 60 to 70 years, particularly for those with low back and waist pain and sciatica. Moreover, the number of pain locations was positively correlated with an increase in cognitive impairment. These findings suggest that clinicians and community-dwelling older adults should be aware of appropriate assessment of bodily pain characteristics and cognitive impairment. We believe that the results of this study help researchers and clinicians who study or treat chronic pain patients with suspected dementia. Pain and cognitive decline need to be simultaneously assessed with much more precision and objective markers, so the quality of life is maintained as best as possible. Further prospective intervention trials are needed to confirm whether incorporating the effective monitoring and management of bodily pain may improve cognitive impairment among community-dwelling older adults.

## Figures and Tables

**Table 1:** Basic characteristics of the study sample.

Characteristics	Bodily Pain (*n* = 1332)		No Bodily Pain (*n* = 690)		*p* Value
	*n*	(%)	*n*	(%)	
Age (Years)					0.090
60–64.9	747	(56.1)	359	(52.0)	
65–70	585	(43.9)	331	(48.0)	
Gender					<0.001
Male	577	(43.3)	400	(58.0)	
Female	755	(56.7)	290	(42.0)	
Marital status					0.599
Unmarried	32	(2.4)	12	(1.7)	
Married	1061	(79.7)	557	(80.7)	
Others	239	(17.9)	121	(17.5)	
Alcohol drinking					0.235
No	1210	(90.8)	615	(89.1)	
Yes	122	(9.2)	75	(10.9)	
Smoking					0.419
No	1001	(75.1)	507	(73.5)	
Yes	331	(24.9)	183	(26.5)	
Routine physical activity					<0.001
No	502	(37.7)	191	(27.7)	
Yes	830	(62.3)	499	(72.3)	
Body mass index (kg/m^2^)	24.5	(3.1)	24.4	(3.1)	0.327
Waist circumference (cm)	86.3	(9.1)	86.4	(8.9)	0.814
Total cholesterol (mg/dL)	200.8	(35.6)	198.4	(35.7)	0.151
Triglyceride (mg/dL)	119.8	(88.5)	110.4	(59.5)	0.005
HDL cholesterol (mg/dL)	54.0	(13.2)	54.0	(13.9)	0.979
LDL cholesterol (mg/dL)	125.2	(32.4)	124.5	(30.7)	0.621
Fasting glucose (mg/dL)	101.2	(21.7)	104.5	(26.7)	0.006
GFR (mL/min/1.73 m^2^)	100.3	(24.2)	98.0	(23.0)	0.033

Values are expressed as mean ± standard deviation or number (percentage). Abbreviation: GFR, glomerular filtration rate; HDL, high-density lipoprotein; LDL, low-density lipoprotein.

**Table 2:** Proportions and univariable and multivariable logistic regression analyses of bodily pain factors for cognitive impairment development among community-dwelling older adults.

Variables	Cognitive Impairment (*n* = 161)		No Cognitive Impairment (*n* = 1861)		*p* Value	Multivariable Model ^b^		
	*n*	(%)	*n*	(%)		OR	(95% CI)	*p* Value
Bodily Pain					0.005			
No	39	(24.2)	651	(35.0)		1.00		
Yes	122	(75.8)	1210	(65.0)		1.68	(1.15–1.16)	0.008
Number of pain locations					0.017			
0	39	(24.2)	651	(35.0)		1.00		
1	58	(36.0)	615	(33.0)		1.61	(1.05–2.48)	0.029
≥2	64	(39.8)	595	(32.0)		1.75	(1.14–2.68)	0.010
Pain location ^a^								
Articular pain	60	(37.3)	581	(31.2)	0.133	1.30	(0.92–1.83)	0.131
Neck pain	48	(29.8)	502	(27.0)	0.460	1.11	(0.77–1.59)	0.576
Low back and waist pain	63	(39.1)	561	(30.1)	0.021	1.47	(1.04–2.06)	0.028
Sciatica	27	(16.8)	199	(10.7)	0.026	1.66	(1.06–2.59)	0.027
Hemicrania continua	27	(16.8)	259	(13.9)	0.345	1.24	(0.80–1.94)	0.337
Other pain	7	(4.4)	71	(3.8)	0.670	1.20	(0.54–2.69)	0.655

Values are expressed as numbers (percentage). CI, confidence interval; OR, odds ratio. ^a^ An individual may have bodily pain in more than one location. ^b^ The models were adjusted for individuals’ age, gender, marital status, alcohol drinking, smoking, physical activity, body mass index, waist circumference, total cholesterol, triglyceride, HDL cholesterol, LDL cholesterol, fasting glucose, and GFR.

## Data Availability

The detailed information on the program and data access is available from the Taiwan Biobank are not publicly available. The authors confirm that, for approved reasons, some access restrictions may apply to the data underlying the findings. The data used in this study cannot be made available in the manuscript, the supplemental files, or in a public repository due to the Personal Information Protection Act executed by Taiwan’s government, starting in 2012. Requests for data can be sent as a formal proposal to obtain approval from the ethics review committee of the appropriate governmental department in Taiwan.
